# Results From a Physical Activity Intervention Feasibility Study With Kidney Inpatients

**DOI:** 10.1177/20543581221079958

**Published:** 2022-02-26

**Authors:** Kathryn Wytsma-Fisher, Manuel Ester, Stefan Mustata, Theresa Cowan, S. Nicole Culos-Reed

**Affiliations:** 1 University of Calgary, AB, Canada; 2 Foothills Medical Centre, Calgary, AB, Canada; 3 Faculty of Kinesiology, University of Calgary, AB, Canada

**Keywords:** physical activity, mobility, frailty, inpatients, kidney disease

## Abstract

**Background:**

Individuals with end-stage kidney disease requiring dialysis are often physically inactive, resulting in reduced physical functioning, increased frailty, and reduced quality of life. Furthermore, extended hospital stays and frequent readmissions are common, exacerbating health care costs. Physical activity may improve physical functioning, disability, and frailty but is not part of standard care of patients requiring dialysis. Research is required to determine the feasibility of implementing physical function assessments and physical activity programs in kidney inpatients requiring dialysis.

**Objective:**

To assess the feasibility and preliminary efficacy of an early-physical activity intervention (Move More study) in the care of kidney inpatients requiring dialysis. It was hypothesized that the intervention would be feasible with regards to administration and participation, meeting *a priori* feasibility criteria, and that kidney inpatients would benefit from participating.

**Design:**

Pilot study.

**Setting:**

Patient Unit 37, Foothills Medical Center, Calgary, AB, Canada.

**Patients:**

Kidney in-patients receiving dialysis.

**Measurements:**

Feasibility data were collected for recruitment, participation, assessment completion, physical activity completion, and adverse events. Participant and healthcare practitioner (HCP) satisfaction with the intervention was assessed using a questionnaire. Frailty and physical function were assessed by the kinesiologist at baseline and postintervention prior to hospital discharge.

**Methods:**

The study was a single-arm pilot intervention examining feasibility and preliminary efficacy. Kidney inpatients requiring dialysis were recruited to an individualized in-hospital physical activity intervention for the duration of their hospital stay. The intervention was led by a kinesiologist and supported by the clinical care team, including physiotherapists and nurse clinicians. Individualized exercise programs were created for patients to perform daily during their stay. These programs focused on strength, mobility, balance, and general movement and were tailored to each patient’s needs.

**Results:**

Thirty-six percent of eligible patients (n = 23/64) consented to participate in the Move More study, of whom 78% (n = 18/23) completed the intervention. The *a priori* level for consent to participate in the intervention was set at 60%. In addition, the *a priori* level for completion of assessments pre and postintervention was 50%. Ninety-five percent (n = 22/23) of preintervention assessments were completed compared to 65% of postintervention assessments. All participants who completed the survey (100%, n = 14/14) and most of the staff (77%, n = 24/31) reported being satisfied or very satisfied with the program. There were no adverse events related to the intervention. On average, Move More patients demonstrated improvements in frailty status and specific subsets of quality of life.

**Limitations:**

Firstly, as a feasibility study, the research was not powered to address the effectiveness of the intervention and lacked a comparison group to definitively link observed changes to the intervention itself. The voluntary nature of recruitment may have been biased toward ESKD inpatients with above-average motivation and baseline function. Furthermore, the small sample size from a single site limits the generalizability of findings. An additional limitation was the fact that postassessments were missed on a large number of patients, due to them being discharged prior to research staff knowing and being able to complete the assessments. Finally, studying the length of stay across the institution, as opposed to just the individual ward, would provide insight into hospitalization impact for these patients.

**Conclusions:**

The administration of and participation in a physical activity intervention for kidney inpatients requiring dialysis was initially not feasible primarily due to low recruitment and adherence challenges. The study was modified by including a research team member on the unit to increase recruitment efforts and support exercise adherence. The intervention impact includes potentially benefits on frailty and quality of life.

## Abrégé

**Contexte:**

Les personnes atteintes d’insuffisance rénale terminale (IRT) nécessitant une dialyze sont souvent inactives physiquement, ce qui entraîne une réduction du fonctionnement physique, une fragilité accrue et une diminution de la qualité de vie. De plus, les séjours prolongés à l’hôpital et les réadmissions fréquentes sont courants chez ces patients, ce qui accroît les coûts des soins de santé. L’activité physique peut améliorer le fonctionnement physique et réduire les incapacités et la fragilité, mais elle ne fait pas partie des soins courants prodigués aux patients nécessitant une dialyze. Des recherches sont nécessaires pour déterminer la faisabilité de la mise en œuvre d’évaluations du fonctionnement physique et de programs d’activité physique chez les patients hospitalisés atteints de néphropathie nécessitant une dialyze.

**Objectifs:**

Évaluer la faisabilité et l’efficacité préliminaire d’une intervention précoce en matière d’activité physique (l’étude *Move More*) intégrée aux soins prodigués aux patients hospitalisés atteints d’insuffisance rénale et nécessitant une dialyze. L’hypothèse était que l’intervention serait réalisable en ce qui concerne l’administration et la participation, qu’elle répondrait aux critères de faisabilité *a priori*, et que les patients hospitalisés bénéficieraient d’une participation.

**Type d’étude:**

Étude pilote.

**Cadre:**

L’unité de soins 37 du Foothills Medical Center de Calgary (Alberta) au Canada.

**Sujets:**

Des patients hospitalisés recevant des traitements de dialyze.

**Mesures:**

Les données de faisabilité ont été recueillies pour le recrutement, la participation, l’achèvement de l’évaluation, l’achèvement du program d’activité physique et les événements indésirables. La satisfaction des participants et des professionnels de la santé à l’égard de l’intervention a été évaluée à l’aide d’un questionnaire. La fragilité et les fonctions physiques ont été évaluées avant et après l’intervention, et avant la sortie de l’hôpital.

**Méthodologie:**

L’étude était une intervention pilote à bras unique examinant la faisabilité et l’efficacité préliminaire. Les patients hospitalisés atteints d’insuffisance rénale et nécessitant une dialyze ont été recrutés pour une intervention individualisée d’activité physique pendant toute la durée de leur séjour à l’hôpital. L’intervention était dirigée par un kinésiologue et appuyée par l’équipe de soins cliniques qui comprenait notamment des physiothérapeutes et des infirmières cliniciennes. Des programs d’activité physique individualisés ont été créés afin que les patients puissent s’exercer quotidiennement pendant leur séjour. Ces programs étaient axés sur la force, la mobilité, l’équilibre et le mouvement général, et étaient adaptés aux besoins de chaque patient.

**Résultats:**

Trente-six pour cent des patients admissibles (n=23/64) ont consenti à participer à l’étude *Move More*; de ceux-ci, 78 % (n=18/23) ont complété l’intervention. Le niveau *a priori* de consentement à participer à l’intervention a été fixé à 60 % et celui de l’achèvement des évaluations avant et après l’intervention à 50 %. Quatre-vingt-quinze pour cent (n=22/23) des évaluations pré-intervention ont été effectuées, comparativement à 65 % pour les évaluations postintervention. Tous les participants qui ont répondu au sondage (100 %; n=14/14) et la majorité du personnel (77 %; n=24/31) interrogé ont déclaré être satisfaits ou très satisfaits du program. Aucun événement indésirable lié à l’intervention n’a été signalé. En moyenne, les patients de *Move More* ont montré des améliorations de leur état de fragilité et de sous-ensembles propres à la qualité de vie.

**Limites:**

Puisqu’il s’agit d’une étude de faisabilité, la recherche n’avait pas la puissance nécessaire pour évaluer l’efficacité de l’intervention et ne disposait pas de groupe de comparaison permettant d’établir un lien définitif entre les changements observés et l’intervention elle-même. La nature volontaire du recrutement pourrait avoir favorisé les patients hospitalisés atteints d’IRT ayant une motivation et une fonction de base supérieures à la moyenne. L’échantillon de petite taille, provenant d’un seul site, limite la généralisabilité des résultats. De plus, les évaluations postintervention ont été manquées pour un grand nombre de patients, ceux-ci ayant obtenu leur congé avant que le personnel de recherche ait été avisé ou en mesure de compléter les évaluations. Enfin, le fait de connaître la durée du séjour dans l’ensemble de l’établissement, et non seulement dans chaque service permettrait de mieux comprendre l’impact de l’hospitalization pour ces patients.

**Conclusion:**

L’exécution d’une intervention d’activité physique et la participation des patients hospitalisés nécessitant une dialyze à une telle intervention n’étaient pas réalisables initialement, principalement en raison de difficultés liées au recrutement et à l’observance. L’étude a été modifiée pour intégrer un membre de l’équipe de recherche dans l’unité de soins afin d’accroître les efforts de recrutement et de soutenir l’observance du program d’exercices. L’impact de l’intervention comprend des avantages potentiels sur la fragilité et la qualité de vie.

## Introduction

Chronic kidney disease (CKD) prevalence is now as high as 71.9 per 1000 individuals in Canada.^1^ Chronic kidney disease is associated with high morbidity, mortality, and poor clinical outcomes, leading to challenges for patients, families, and health care providers.^2^ As CKD progresses to kidney failure, these issues are exacerbated. Individuals requiring dialysis are often physically inactive, have reduced physical abilities and difficulties performing routine daily tasks, lower health-related quality of life (QOL), and higher cardiovascular morbidity and mortality compared to the general population.^3-5^ As a result of this disease burden, kidney patients, especially those on dialysis, have high hospitalization rates.^6^ Due to frailty and reduced physical function, many are unable to return home once admitted to hospital, resulting in prolonged hospital stays as well as frequent readmissions to hospitals or transition to long-term care facilities.^7^ This results in substantial healthcare costs associated with inpatient care.^2,7^

The prevalence of frailty and limited physical function in kidney patients requiring dialysis is high from deconditioning and increased muscle wasting.^8^ More than 60% of kidney patients are considered frail, compared to only 11% of older adults.^9^ In dialysis patients, frailty is common and associated with adverse outcomes^10^ such as poor physical functioning.^11^ Frailty increases risk of health complications such as falls, cardiovascular events, hospitalization, disability, reduced QOL, and mortality.^12-15^ Therefore, the management of frailty is central to kidney care.

Physical activity interventions show promise for managing frailty in geriatric populations.^16^ A systematic review of physical activity on frailty found high adherence and no adverse events in most studies, concluding that physical activity is safe and feasible for an aging population, with greater impact during early stage frailty.^16^ Additionally, physical activity can increase strength, physical performance and muscle mass in CKD patients, all of which are critical to combating frailty.^17^ As physical activity interventions demonstrate efficacy for treating and preventing frailty and disability in frail elderly individuals, similar interventions are likely to benefit CKD patients, who share many symptoms with this population.^17^

Evidence highlights the value of including physical activity in standard late stage kidney care.^18^ Physical activity can improve blood pressure,^12,19^ insulin sensitivity, maximal exercise capacity, physical performance and self-reported functioning^8^ in kidney patients. Additionally, physical activity has been shown to increase QOL and cognitive function while reducing symptoms of depression.^12^ Evidence from other populations suggests that physical activity interventions for critically ill patients can enhance functional capacity, strength, mobility, and QOL, reduce length of stay (LOS), and increase the probability of being discharged home.^20^

Research is needed to identify optimal strategies to implement physical function assessments and promote physical activity in patients with kidney patients requiring dialysis.^8^ The purpose of this study was to assess the feasibility and preliminary efficacy of an early-physical activity intervention in kidney inpatients requiring dialysis care. It was hypothesized that both administration of and participation in the intervention would be feasible. Secondary outcomes were collected to assess potential benefits of the program on physical, functional, and patient-reported outcomes.

## Methods

### Study Design

The Move More study was a single-arm feasibility study examining intervention feasibility and preliminary efficacy. The study protocol has been described in detail previously.^21^ Ethical approval was obtained through the Conjoint Research Ethics Board of the Faculties of Medicine at the University of Calgary (REB 18-2134).

### Recruitment and Eligibility

Eligibility criteria included: (1) kidney inpatients on Unit 37 (inpatient nephrology and transplant) on maintenance dialysis (hemodialysis or peritoneal dialysis), (2) anticipated date of discharge (ADOD) greater than 7 days, and (3) deemed medically stable to participate in physical activity by a nurse or nurse clinician. Exclusion criteria due to medical instability were defined as (1) unstable vital signs, poor oxygen saturation on room air, (2) decreased level of consciousness, confusion, (3) febrile (temperature >38.3°C), (4) chest pain or shortness of breath at rest, (5) signs of gastrointestinal (GI) bleed, (6) pain which can be potentially exacerbated by physical activity intervention, or (7) a permanent negative change in medical status (ex. change to palliative care). Patients remained enrolled if there was a temporary decline in medical status that prevented participation as long as the patient was expected to recover. Owing to the variability in medical care for individuals on maintenance dialysis, patients remained enrolled in the study in the case of a temporary transfer off the renal inpatient ward for medical procedures on another ward. Patients who were permanently transferred to another ward were discharged from the study.

### Study Procedure

Eligible inpatients were identified by nurse clinicians or the research coordinator using the electronic medical record (EMR), before being approached by clinical staff for consent to contact from the research team for further information. Those who agreed to participate signed an informed consent form before completing a baseline assessment and instructional session on performing tailored exercises with a kinesiologist. In response to slow recruitment rates initially, a research staff member was stationed on the ward several days a week, resulting in improved patient recruitment and study awareness with the staff.

### Physical Activity Intervention

Inpatients received an individualized physical activity intervention according to baseline measures. Patient mobility was determined by a review of charts and nursing/physiotherapy (PT) notes to determine level of assist: (1) bed-bound (2-person assist/full lift); (2) moving around room (1-person assist); and (3) moving out of room (independent), each with its own starting template for exercises. Physical activity was tailored based on phase and participant preferences, with few standing exercises performed due to a high risk of falls and limited space within the patient’s rooms.

Participants were instructed to complete prescribed physical activity daily and encouraged to progress as determined by the kinesiologist. Strength exercises were selected from the Vivifrail Multicomponent Physical Exercise Program, which has shown positive effects in elderly inpatients.^22^ A target intensity of 2-3 on the 10-point Borg rating of perceived exertion scale was used, which is considered best practice in advanced renal disease settings.^23,24^ In addition, participants were encouraged to perform physical activity beyond their daily prescription as tolerated. The intervention exercises can be seen in Table 1. The kinesiologist would select 3 strength exercises for each patient, adding more if they were well-tolerated by the patient. Exercises were also based on patient preferences, thus not all patients would complete both strength and aerobic exercises. The planned frequency of exercise was 2 sessions per week with the kinesiologist, with other staff (including PT, nursing, and health care aides) performing some activity with patients daily. Kinesiology sessions were around 30 mins. Usual care from occupational therapy and PT were continued for each patient who had acute care goals in addition to any kinesiology-based exercise. Not all participants participated in physio.

**Table 1.** Overview of Foundational Exercise Intervention.

Phase	Phase	Resistance exercises	Aerobic exercises
1	Bed-bound or 2-person assist	5 mins or more of selected exercises with or without resistance bands: Chest press, rows, biceps curl, triceps extensions, glute squeeze, knee extensions/heel slide, calf raises—seated or ankle pumps, supine hip abduction.	5 mins or more, bed bike
2	Moving around room, 1-person assist	10 mins or more of selected exercises with or without resistance bands: Chest press, rows, biceps curl, triceps extensions, glute squeeze, knee extensions, calf raises- seated or standing, standing hip abduction, seated hip abduction, hamstring curl seated or standing, quarter squat.	10 mins or more, bed and/or leg bike
3	Moving out of room, independent	15 mins or more of selected exercises with or without resistance bands: Chest press, rows, biceps curls, triceps extensions, glute squeeze, knee extensions, calf raises- seated or standing, standing hip abduction, seated hip abduction, hamstring curl seated or standing, quarter squat, sit to stand.	15 mins or more, leg bike and/or walking

### Primary Outcomes

The primary outcome of the study was feasibility of administering and participating in an individualized physical activity intervention, as determined using *a priori* feasibility criteria. Predetermined feasibility criteria included 60% recruitment rate, 50% assessment completion, no adverse events related to the intervention, and high satisfaction (a mean score of 4/5 on the Likert-type scale) as reported by both the participants and HCPs. These criteria were set based on clinical consultation and examination of previous literature.^25-27^ Recruitment included measures of eligibility, numbers approached, numbers consented, and total numbers participating. Next, completion of the prescribed exercise/mobility intervention as per records on the EMR was assessed. Participant and HCP satisfaction with the exercise intervention, measured by satisfaction surveys, was examined along with HCP completion of clinical charting for FITT prescription (date, frequency, intensity, duration, type of exercise; progression through strengthening exercises) and level of assist needed (if any). Lastly, participant assessment as well as reporting of adverse events related to participation in the in-hospital exercise program were examined.

### Secondary Outcomes

Secondary outcomes included frailty, as measured using the modified Fried Frailty Index,^28^ functional measures,^29^ QOL, as measured by the Kidney Disease Quality of Life Instrument (KDQOL),^30^ number of falls in-hospital, hospital LOS, and 30-day readmissions. Frailty, functional measures, and QOL were assessed at baseline and postintervention prior to hospital discharge. Hospital LOS, 30-day readmissions and number of falls during the intervention were assessed via EMR review. Measurement for all secondary outcomes have been described previously;^21^ however, the values used for the Fried Frailty Index are provided in Table 2. Physical activity was assessed with the Godin Leisure Time Exercise Questionnaire (GLTEQ), and a physical activity score was calculated based on the weekly activity score and the amount of strenuous, moderate, and mild activity.^31^ The score (in units) is classified as active (24 units or more), moderately active (14-23 units), and insufficiently active (less than 14 units).^31^

**Table 2.** Fried Frailty Index Scoring Values.

GLTEQ			Fried score
Active	≥24 points		0
Moderately active	14-23 points		0
Insufficiently active	<14 units		1
Grip strength	BMI	Grip strength	Fried score
*Men*			
	BMI < 24	<29 kg	1
	BMI 24.1-26	<30 kg	1
	BMI 26.1-28	<30 kg	1
	BMI > 28	<32 kg	1
*Women*			
	BMI < 23	<17 kg	1
	BMI 23.1-26	<17.3 kg	1
	BMI 26.1-29	<18 kg	1
	BMI > 29	<21 kg	1
Gait speed	Height	Time cut off	Fried score
*Men*			
	<173 cm	>7 s	1
	Height > 173 cm	>6 s	1
*Women*			
	<159 cm	>7 s	1
	>159 cm	>6 s	1
Fried categories	Total score (sum of all 5 categories)		
Not frail	0-1		
Prefrail	1-2		
Frail	≥3		

*Note.* If the patients grip strength is below the cut off for their given BMI, then the person receives a 1 for this component of the Fried Frailty Index. If their grip strength is above the cut-off, then the person will receive a 0. If the patient’s gait speed is slower than the cut off, they receive a 1 for Fried phenotype. If it is faster than the cut-off, they receive a 0. If the patient’s self-reported exhaustion score is positive, they receive a score of 1 for this category. If the patient’s self-reported unintentional weight loss is ≥10 lbs, they receive a score of 1 for this category. GLTEQ = Godin Leisure Time Exercise Questionnaire; BMI = body mass index.

### Sample Size

All kidney inpatients requiring dialysis admitted to the nephrology inpatient service at the Foothills Medical Center in Calgary requiring dialysis and meeting the inclusion criteria were approached for participation in the feasibility study. As a feasibility study, *a priori* sample size calculation was not performed. After conversations with the Unit 37 manager and the average number of patients referred to the kinesiologist prior to the intervention (n = 8-10 per month), 24 to 36 patients were expected to be recruited over 12 months. It was anticipated that only n = 2 to 3 patients (out of the n = 8-10 referred to the kinesiologist) would meet the study inclusion criteria of ADOD > 7 days.

### Statistical Analysis

Participant demographics and outcomes were summarized using descriptive statistics. For continuous variables, means and standard deviation were calculated. For categorical variables, frequencies and percentages were presented. Within-group changes in secondary outcomes of frailty, PA, QOL, and physical function were examined using dependent sample *t*-tests (continuous, normally distributed) or Wilcoxon signed-rank tests (categorical, nonnormally distributed) depending on the nature of the variable. Descriptive statistics were used to provide an overview of additional measures for LOS, in-hospital falls, and readmission. All analyses were completed using SPSS statistics, version 26.0 (IBM).

## Results

### Participant Characteristics

**Figure .1:**
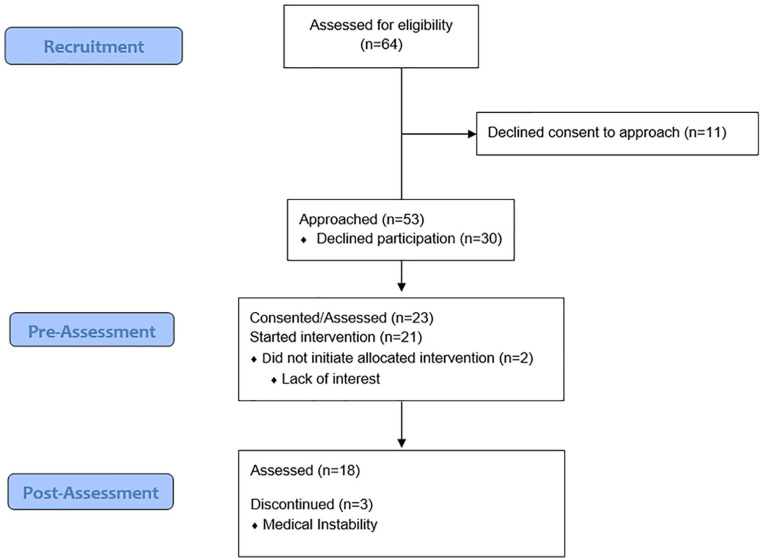
Study overview flow diagram.

There were a total of n = 23 participants, 14 male and 9 female, with an average body mass index (BMI) of 28.6 kg/m^2^ ± 5.21 kg/m^2^ (Figure 1). Most participants were on hemodialysis (n = 16/22) with the main cause for dialysis being diabetes and/or hypertension (n = 12/16). Reason for admission to hospital and dialysis vintage information are outlined in Table 3. At baseline, most participants were insufficiently active (53%, n = 9/17) according to the GLTEQ (Table 3).

**Table 3.** Participant Demographics.

Variable				
Sex	Male	Female		
	14	9		
Age (years)				
Mean (SD)	63.6 (15.3)			
BMI (kg/m^2^)				
Mean (SD)	28.6 (5.2)			
Admission Diagnosis	Musculoskeletal	Infection	Kidney related	Other
	6	4	5	7
Type of dialysis	Hemodialysis	Peritoneal dialysis		
	16	6		
Dialysis vintage (months)				
Mean (IQR)	46 (32)			
Cause of dialysis	Diabetes/hypertension	Focal segmental glomerulosclerosis	Glomerulonephritis	Other
	12	3	2	5
Physical activity at baseline (GLTEQ)	Active	Moderately active	Insufficiently active	
	5	3	9	
Level of assist	2-person assist/full lift	1-person assist	Independent	
	7	5	9	

*Note.* BMI = body mass index; GLTEQ = Godin Leisure Time Exercise Questionnaire; IQR = interquartile range.

## Feasibility

### Recruitment

Over 8-months, weekly reviews of inpatient files identified 64 potential participants. Recruitment to the Move More intervention was 36% (n = 23/64), lower than the *a priori* set recruitment level of 60%. Two participants signed the informed consent but withdrew before starting the intervention, due to lack of interest. Although the recruitment rate of 36% fell below the predicted 60% threshold, patient recruitment was enhanced midway into the study by the presence of a research team member on the ward. The research team member was better able to explain the study to patients as well as remind staff about consent to approach for eligible patients. The study began in August 2019 and by October 2019, the recruitment rate was 26% (8 enrolled, 31 screened). A research team member was introduced at this point, and by the end of January 2020, the recruitment rate had risen to 50% (10 enrolled, 20 screened) with a total recruitment rate of 35% (18 enrolled and 51 screened) (Table 4).

**Table 4.** Feasibility Measures.

Recruitment		N	%	*A priori* level
	Eligibility	64		
	Consented to approach	53	83	
	Consented to intervention	23	36	60.0
	Completed intervention	18	28	
Assessment completion	Preintervention	22	96	50.0
	Postintervention	15	65	50.0

### Completion

Twenty-two participants completed the preintervention consent and assessment, 21 started the intervention, and 78% of those who consented (n = 18) completed the intervention, compared to the predicted 50% *a priori* level. The 3 participants who did not complete the intervention were withdrawn by research staff due to medical instability.

On average, participants completed physical activity on 55% (n = 447/808) of intervention days. This was determined by records of activity on the EMR and did not include patient self-reported sessions. Reasons for declining exercise participation were not collected.

### Clinical Charting Completion

There were 1101 documented physical activity sessions across all clinicians, including multiple sessions per day for some patients. The healthcare staff who documented the completion of exercise sessions included healthcare aides and the kinesiologist, with 26% (n = 282) and 23% (n = 257) documented sessions, respectively. Nurses documented 21% (n = 228) exercise sessions, with their nursing students documenting an additional 4.8% (n = 53) sessions. PT (n = 175) and therapy assistants (n = 101) documented most of the remaining sessions at 16% and 9.2%, respectively.

### Program Satisfaction

**Figure 2.**
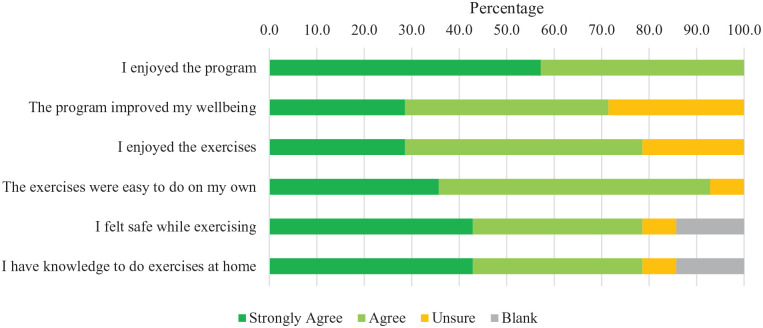
Patient satisfaction.

**Figure 3.**
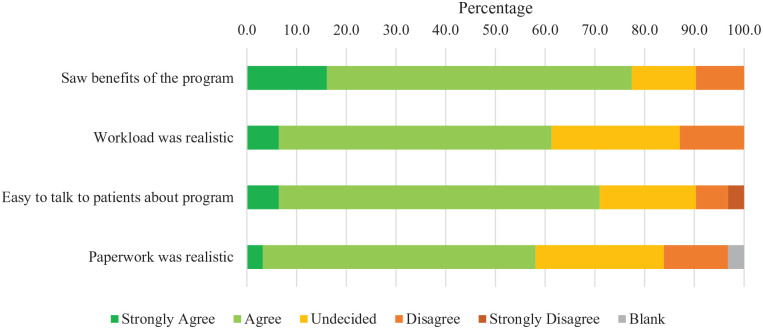
Healthcare provider satisfaction.

Participant satisfaction was high (Figure 2), with 100% of participants who completed the satisfaction survey (n = 14) agreeing or strongly agreeing to the statement “I enjoyed the program.” The other participants (n = 10) who did not complete the survey were either withdrawn (n = 4) from the study or did not do any discharge measurements (n = 6). Most participants (71%) agreed that the program improved their well-being (n = 10/14), 79% enjoyed the exercises (n = 11/14), and 79% felt safe while exercising (n = 11/14). Similarly, staff reported satisfaction with the intervention. Most staff (68%) “agreed” or “strongly agreed” (n = 21/31) that they saw the benefits of the program (Figure 3). In addition, 61% of staff found that the workload for the study was realistic (n = 19/31), and 71% felt they could talk to patients about the study (n = 22/31).

### Assessment and Adverse Events

Physical function assessments were completed on 96% of participants at baseline (n = 22/23) and 65% of participants postintervention (n = 15/23), higher than the *a priori* set 50% completion levels. Assessments were not completed for 7 participants due to hospital discharge prior to assessment completion. There were no adverse events related to the intervention.

## Secondary Outcomes

**Figure 4.**
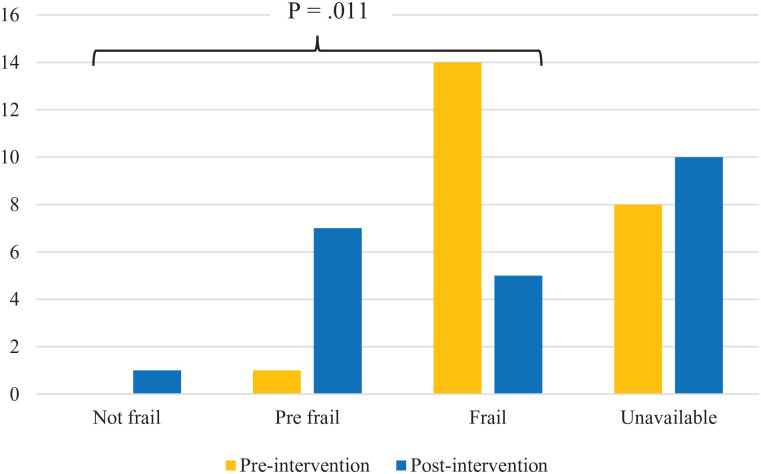
Preintervention and postintervention Fried frailty scores. *Note.* Paired samples N = 11. The *P* value is based on the outcome of the Wilcoxon Signed-Rank Test for change in frailty scores. Quality of Life.

Owing to room size limitations and risk of falls, walking tests could not be completed. Therefore, Fried frailty was measured using exhaustion, weight loss, and grip strength. Based on the modified Fried frailty index, frailty improved significantly from preintervention to postintervention (*P* = .011). As seen in Figure 4, several participants improved from frail to prefrail after completing the PA interventions. At baseline, 14/23 participants were frail, and 1/23 participants were prefrail. After the intervention, 5/23 were frail, 7/23 were prefrail, and 1 participant was notfrail. In addition, after the intervention, only 29% (n = 4/14) of participants were insufficiently active, while the remaining 71% (n = 10/14) were classified as moderately active or active (Table 3).

**Figure 5.**
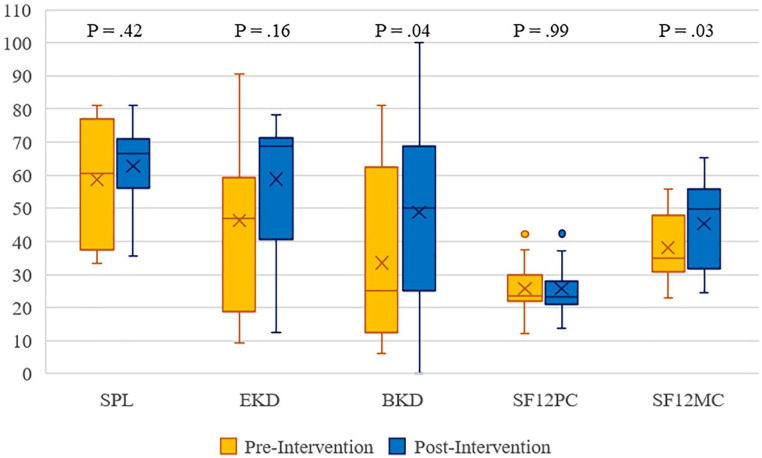
Quality of life pre/postintervention according to KDQOL-36. *Note.* KDQOL = Kidney Disease Quality of Life Instrument; SPL = symptom/problem list; EKD = effects of kidney disease; BKD = burden of kidney disease; SF12PC = SF-12 physical composite; SF12MC = SF-12 mental composite.

As demonstrated in Figure 5, 2 KDQOL sections improved over the course of the study. The burden of kidney disease score improved (33.5 ± 25.5 to 48.9 ± 27.5, *P* = .037), as did the mental composite score (38.0 ± 10.8 to 45.3 ± 13.7, *P* = .031). The remaining KDQOL sections did not change over the course of the intervention.

The average LOS for participants in the study was 71.9 ± 61.2 days in hospital. Seven participants were readmitted to hospital within 30 days of their initial discharge. The reasons for readmission are outlined in Table 5.

**Table 5.** Healthcare Utilization.

N = 22	Mean	SD	Median
Length of stay (days)	71.9	61.2	53
	N	%	
30-day readmission	7	30	
Readmission reasons	Bleeding n = 3 Infection n = 1 Other n = 3		

## Discussion

The Move More study introduced a physical activity intervention into standard kidney care in-hospital. During the study period, 63 patients were determined to be eligible, and 88% of those (n = 54) consented to be approached by a research team member. However, only 36% of patients approached consented to the intervention, compared to the *a priori* level of 60%. Low recruitment rates for patients are common in exercise interventions for CKD patients.^32^ While a previous walking program^32^ was found to be feasible without additional staff or resources, the authors stated how recruitment into the program was difficult and time consuming. Part-way through the intervention, a research team member was placed on the ward to focus on the study. This helped to increased uptick in eligible patient identification. Even with a team member present, the recruitment rate of 50% falls below the threshold of 60%. While the team member greatly enhanced the recruitment (+24%), there are still further improvements needed to reach the 60% goal for feasibility. Alternatively, given the acuity of the inpatients on Unit 37, 60% may not be achievable. Assessment completion rates (80%) exceeded the 50% threshold set *a priori*, and there were no adverse events related to the intervention. Satisfaction with the program was excellent among both participants and HCPs. Overall, other than lower than anticipated consent rates, the Move More study was feasible when there was direct research support established on the ward.

Beyond feasibility, the implementation was also examined and included the role of the kinesiologist on this inpatient study. Throughout this study, the kinesiologist was intended to support the clinical staff in physical activity services, allowing for consistent monitoring and adaptation of programming to enhance safety, adherence, and intervention effectiveness. Having a kinesiologist, with the role of aiding recruitment and promoting movement while in-hospital, as part of the care team on the ward did result in more mobility and engagement in exercise. Unlike the previous study,^33^ where exercise was performed while on hemodialysis, Move More participants exercised while on the inpatient ward. The benefit of having participants exercise while on the ward is that hemodynamic stability would have been required for exercising on dialysis, limiting the participation of some individuals. Additionally, a kinesiologist on the ward allowed for more variety of exercises, such as strength, walking, and biking, and exercise choice was tailored based on patient preference. The kinesiologist also provided the supervision to ensure that the patient felt supported. Normally, the care team of nurses and PT have significant caseload requirements and would not have the time to individualize exercise programming or provide that tailored support. As well, most PT focus on acute care goals. Finally, having a kinesiologist on the ward was important to promote physical activity with patients and kept them accountable to repeat the exercises on their own, when the kinesiologist was not present. The substantial interaction between the kinesiologist and patients was reflected in the physical activity sessions, with 23% performed under the supervision of the kinesiologist. As in previous studies,^34^ a key to ongoing success of integrating mobility in patients is the documentation of activity requirements into the care plan documents. This is critical to ensure all staff are aware of mobility expectations and can work to get patients moving. It is important to note how much the Move More intervention relied on staff, including support from management, adding mobility to comfort rounds and flow sheets, as well as completing frequent audits that physical activity was being entered, separate from the study.

### Secondary Outcomes

The Move More intervention had positive impacts on frailty. At baseline, 14/23 patients were considered frail. At discharge, 7 of those 14 patients were either prefrail or not frail. Improving frailty is critical in this population. Sedentary behavior and minimal physical activity, which contributes to poor physical functioning and frailty, is seen in many hemodialysis patients.^35^ Poor physical performance/function and frailty is also associated with elevated risk of hospitalization, death, and disability.^11,36^ As a result, more healthcare dollars are spent per patient and worsening patient outcomes are seen.

Some patients saw improvements in QOL over the course of this study. Although patient QOL scores from baseline to the end of the study may have been impacted as their health improved throughout the course of the hospital stay, the integration of regular physical activity into their routines may have also contributed. Regular physical activity improves both physical and psychological aspects of health in kidney patients, which are related to key outcomes such as mortality.^12^

### Limitations

There are several limitations to this study. First, many postintervention assessments were missed for patients, due to discharges happening when research staff were not present on the unit. This makes it difficult to fully assess change from baseline and highlights the importance of timely and ongoing communication between the research team and the clinical staff. Second, as a feasibility study, the research was not powered to address the effectiveness of the intervention and lacked a comparison group to definitively link observed changes to the intervention itself. Rather, this feasibility work falls under the category of informing process and determining recruitment, retention, and adherence.^26^ Third, the voluntary nature of recruitment may have been biased toward ESKD inpatients with above average motivation and baseline function. Furthermore, the small sample size from a single site limits the generalizability of findings. Important to note was that patients who were permanently transferred to another ward were discharged from the study (n = 2). Kidney patients generally have long hospitalization stays which strain healthcare systems, as well as being hard on the patients themselves.^6^ Studying the LOS across the institution would provide insight into hospitalization impact for these patients.

## Conclusion and Future Work

The implementation of a physical activity intervention for kidney inpatients requiring dialysis was initially not feasible, primarily due to challenges including low recruitment and adherence to the exercise regime. The modification of including a research team member on the unit was critical for increasing recruitment rate from 26% to 35%. Given this remained below the predetermined level of 60% for recruitment feasibility, additional work must continue to implement modifications to support movement and mobility efforts for the patients while on the unit. The role of the kinesiologist, linking the research and clinical priorities by supporting both recruitment and patient exercise adherence, is critical for future implementation research. Finally, patient and staff satisfaction was high throughout the intervention. In addition to these feasibility markers, the intervention also showed early promise for impacting frailty status and some aspects of QOL for participants.

To better understand the impact of a physical activity intervention in kidney inpatients requiring dialysis, a larger, appropriately powered trial that includes a comparison arm is warranted. Furthermore, future work should explore the barriers to recruitment and participation in exercise programs among kidney in-patient populations. The potential benefits of physical activity, including those on function and QOL, must be available to kidney patients by building programs into standard inpatient care.
